# Biodegradable Gelatin–Carrageenan Sponges: High-Potential Functional Nasal Packs for Efficient Secretome Delivery

**DOI:** 10.3390/polym16233387

**Published:** 2024-11-30

**Authors:** Rabiatul Adawiyah Razali, Fairuz Izan Muhammad Firdaus, Mh Busra Fauzi, Nadhratun Naiim Mobarak, Saim Aminuddin, Yogeswaran Lokanathan

**Affiliations:** 1 Medical Innovation Research Centre (MIRC), Shiga University of Medical Science, 520-2192 Otsu, Shiga, Japan; rabiatularzl@gmail.com; 2 Department of Tissue Engineering and Regenerative Medicine (DTERM), Faculty of Medicine, Universiti Kebangsaan Malaysia, Kuala Lumpur 56000, Malaysia; firdaus.dann@gmail.com (F.I.M.F.); fauzibusra@ukm.edu.my (M.B.F.); 3 Department of Chemical Sciences, Faculty of Science and Technology, Universiti Kebangsaan Malaysia, Bangi 43600, Selangor, Malaysia; nadhratunnaiim@ukm.edu.my; 4 Graduate School of Medicine, KPJ Healthcare University, Kota Seriemas, Nilai 71800, Negeri Sembilan, Malaysia; aminuddin_saim@yahoo.com; 5 Advance Bioactive Materials-Cells UKM Research Group, Universiti Kebangsaan Malaysia, Bangi 43600, Selangor, Malaysia

**Keywords:** blood absorption, biomaterial, wound healing, drug delivery, wound pack

## Abstract

Nasal packing is a critical procedure in postoperative care and trauma management aimed at controlling bleeding, providing structural support, and promoting tissue healing. However, conventional nasal packs often lead to discomfort, infection risks, and secondary tissue damage. To address these challenges, this study explores the potential use of biodegradable and biocompatible gelatin–carrageenan composite scaffolds as an alternative nasal packing material. Five compositions of gelatin–carrageenan scaffolds (ratios 10:0, 7:3, 5:5, 3:7, and 0:10) were fabricated and evaluated for physicochemical properties, hemocompatibility, and cytocompatibility. Results suggest that balanced ratios, such as 7:3 and 5:5, may provide a combination of structural integrity, improved biocompatibility, and controlled degradation, making them a potential candidate for nasal packing applications. The scaffolds exhibited low cytotoxicity and reasonable blood compatibility, which could reduce the risks associated with conventional materials. While these findings are promising, further in vivo studies are necessary to validate the efficacy and safety of these scaffolds in clinical settings. If proven effective, gelatin–carrageenan scaffolds may help address some of the limitations of conventional nasal packing materials and improve postoperative care outcomes.

## 1. Introduction

Nasal packing is a widely used medical intervention to control bleeding, stabilize wounds, and support tissue healing following nasal surgeries or traumatic injuries. However, while effective at achieving hemostasis, conventional nasal packing materials often have limitations that impact patient comfort and recovery. Issues such as adhesion formation, granulation, infection risk, and discomfort during removal highlight the urgent need for biocompatible materials that can enhance patient outcomes while minimizing these drawbacks [1,2,3].

There are two primary types of nasal packing: absorbable and non-absorbable. Absorbable nasal packs are designed to degrade in situ, reducing the need for removal and, consequently, decreasing discomfort and mucosal damage [4,5]. Conversely, non-absorbable packs require removal, which can lead to patient anxiety, secondary mucosal injury, and an increased risk of infection [6,7]. A current systematic review shows an ongoing debate regarding the advantages of these types, with absorbable packs offering greater comfort. In contrast, non-absorbable packs provide stronger mechanical support and better control over bleeding and edema [8,9,10]. Therefore, fabricating a new nasal packing material necessitates careful consideration of both functionality and material composition to achieve an optimal balance between patient comfort and clinical efficacy.

A promising approach to address these challenges is the use of biodegradable and biocompatible materials such as gelatin and carrageenan [10]. Gelatin is a biopolymer derived from collagen through a hydrolysis extraction process. This process produces a soluble protein with distinctive functional properties, including its ability to gel and thicken [11,12]. Gelatin also has proven effective for nasal packing, especially post-surgery, due to its hemostatic properties, biocompatibility, and support for wound healing [13,14]. Unlike traditional materials that may lead to discomfort upon removal [15], gelatin sponges like Gelfoam are absorbable, reducing the need for removal and minimizing the risks of rebleeding and scarring [16,17]. In addition, studies show gelatin promotes epithelialization, decreases complications such as synechiae, and retains moisture, supporting recovery in delicate nasal tissues [18,19]. However, while generally safe, gelatin may carry minor risks in specific cases [20], underscoring the need for careful postoperative monitoring.

Carrageenan, a polysaccharide extracted from red seaweed, offers hydrophilicity, tunable degradation, and enhanced mechanical strength [21]. Carrageenan is used in biomedical applications due to its favorable water retention, bioactivity, and gelation properties [22,23]. One of the primary benefits of carrageenan in nasal applications is its antiviral activity. Research has demonstrated that carrageenan can inhibit various respiratory viruses, including rhinoviruses and influenza viruses, by forming a protective barrier on the nasal mucosa [24,25,26]. Moreover, carrageenan’s mucoadhesive properties make it particularly suitable for nasal applications. When used in nasal packs, carrageenan can adhere to the nasal mucosa, providing prolonged contact time and enhancing the delivery of therapeutic agents [27]. This characteristic is crucial for ensuring that the antiviral effects of carrageenan are maximized, as it allows for sustained release and activity against pathogens present in the nasal cavity [28]. Additionally, the mucoadhesive nature of carrageenan can help maintain moisture in the nasal passages, which is beneficial for patients recovering from nasal surgeries or those suffering from chronic nasal conditions [29].

Composite scaffolds with tailored mechanical, physical, and biological properties can be developed by combining gelatin and carrageenan, making them highly suitable for nasal packing applications. Gelatin is advantageous for its biocompatibility, hemocompatibility, and absorbability; however, it presents challenges related to mechanical strength, moisture retention, and the potential for discomfort upon removal. The incorporation of carrageenan is expected to address these limitations effectively while also contributing valuable antiviral and mucoadhesive properties, thereby enhancing the overall functionality and comfort of the scaffold.

This study investigates the fabrication and characterization of gelatin–carrageenan scaffolds with varied compositions to determine the optimal material properties for nasal packing applications. By examining properties such as porosity, swelling, water vapor transmission, hemocompatibility, and cytocompatibility, this research aims to develop an advanced nasal packing material that balances structural integrity, biocompatibility, and hemostatic performance. Findings from this study are expected to contribute to safer, more effective nasal packing technologies, improving patient outcomes through reduced complications and enhanced healing. 

## 2. Methodology

This study received approval from the Universiti Kebangsaan Malaysia Research Ethics Committee (UKM PP/11/8/JEP-202-509). All methodologies and experimental procedures adhered strictly to the guidelines and regulations stipulated by the committee.

### 2.1. Gelatin–Carrageenan Sponge Fabrication

A 10% (*w*/*v*) genipin solution was prepared by dissolving genipin in 70% ethanol for stock. A 1% (*w*/*v*) gelatin (Nitta-gelatin, Osaka, Japan) solution was prepared with Dulbecco’s Phosphate-Buffered Saline (DPBS) and stirred for 30 min at 40 °C. The solution was then kept at 37 °C to prevent solidification until use. A 1% carrageenan solution was prepared by stirring carrageenan powder with DPBS at 70 °C until dissolved. From there, 5 groups of different concentration ratios of gelatin: carrageenan were prepared (10:0, 7:3, 5:5, 3:7, and 0:10). Genipin solution stock was introduced to the mixture to achieve a 1% final genipin concentration. Subsequently, 2 mL of the mixture was quickly added to a round silicone mold. The scaffolds were left to set and crosslinked at room temperature for 24 h, then pre-frozen at −20 °C overnight, and then lyophilized for 48 h. Scaffolds were kept in a petri dish inside a sealed tight container with desiccant beads at room temperature until used. All experiments were performed in a set of 5 ratios and will be referred to as scaffolds.

### 2.2. Field Emission Scanning Electron Microscope (FESEM) and Pore Size

The morphology and porosity of the scaffolds were examined using the field emission scanning electron microscope (FESEM) (Zeiss/Supra 55VP, Oberkochen, Germany). Scaffolds were cut into two to expose the inner side of the scaffolds. Prior to FESEM viewing, the lyophilized scaffolds underwent gold coating. The scaffolds were observed at a magnification of 100× and 30×. The average pore size was determined from FESEM images of scaffolds obtained using Image J software v 1.54 (Madison, WI, USA).

### 2.3. Porosity

The porosity of the scaffolds was assessed using the liquid displacement method with ethanol. The volume of each scaffold was calculated using the formula *V* = *πr*2h for cylindrical scaffolds and *V* = *a*^3^ for cubic scaffolds. The initial weight of the dry scaffolds (*Wi*) was recorded. Each scaffold was placed in a well plate, and pure ethanol was added until the scaffolds were completely submerged. The samples were incubated at room temperature for 24 h. After incubation, the final weight of the scaffolds (*Wf*) was recorded. The porosity of the scaffolds was determined using the following formula: Porosity (%) = ((*Wf* − *Wi*)/ρV) × 100% where ρ is the density of ethanol (0.78945 g/cm), and *V* is the volume of the scaffold. 

### 2.4. Fourier Transform Infrared Spectroscopy (FTIR)

FTIR spectra were obtained using a Perkin Elmer Spectrum 400 FTIR Spectrometer with wavenumbers ranging from 400 to 4000 cm^−1^ (PerkinElmer, Shelton, CT, USA). Spectral data were analyzed using built-in software to identify characteristic molecular fingerprints, ensuring accurate determination of the scaffolds’ chemical compositions.

### 2.5. Hydrolytic Degradation

Scaffolds were prepared and weighed before being immersed in a 0.0006% (*w*/*v*) collagen type I solution containing sodium azide (to prevent microbial contamination). Every 24 h, the scaffolds were carefully removed, blotted dry to remove excess solution, and weighed using an analytical balance to monitor weight loss due to degradation. 

### 2.6. Swelling and Absorption Rate

Scaffolds were placed in a 24-well plate, and their initial weights (Wd) were recorded. Phosphate-buffered saline (PBS) was added to each well until the scaffolds were fully soaked, and the plates were left at room temperature for 2 min, 30 min, and 6 h. The weights of the scaffolds were then recorded at every interval (Ww). The swelling ratio was calculated using the formula: Swelling Ratio (%) = ((Ww − Wd)/Wd) × 100, where Ww is the wet weight and Wd is the dry weight. The absorption rate graph was plotted using the weight obtained from every interval.

### 2.7. Compression and Resilience

Each scaffold was placed on a flat surface, and a photo from the side profile was taken prior to compression. A constant weight of 300 g was applied to the scaffold for 5 min, followed by another side-profile photo to document the scaffold after compression. Subsequently, the scaffold was immersed in distilled water for 5 min, and a final photo was taken after hydration. The areas of the scaffold in each condition were measured using ImageJ software. The compression ratio (C%) was calculated using the formula C% = ((*Ai* − *Ac*)/*Ai*) ×100, where *Ai* is the initial area and *Ac* is the area after compression. The resilience ratio (R%) was calculated using the formula: R% = ((*Af*/*Ac*)) ×100, where *Af* is the area after hydration.

### 2.8. Contact Angle

The contact angle of the scaffolds was measured by placing a 5 µL droplet of distilled water on the scaffold surface and capturing an image of the droplet from the side. ImageJ software was then used to analyze the contact angle, which is defined as the angle formed between the liquid droplet and the solid scaffold surface at the contact point.

### 2.9. Water Vapor Transmission Rate (WVTR)

The water vapor transmission rate (WVTR) was measured using a setup with Bijou bottles and distilled water. The area of the bottle opening was measured, and 10 mL of distilled water was added to each bottle. The intended scaffold was placed on the opening and sealed with parafilm. The initial weight (Wi) of the sealed bottle was recorded. The bottles were then incubated in a 37 °C incubator for 24 h. After incubation, the bottles were weighed again to obtain the final weight (Wf). The WVTR was calculated using the formula: WVTR = ((*Wi* − *Wf*))/(area (m^2^) × day), where Wi is the initial weight, Wf is the final weight, and the area is the opening of the bottle.

### 2.10. Blood Uptake Assay

Blood uptake was measured following the method by Hajosch et al. (2010) [17]. Instead of the 2 cm round scaffold mold, scaffolds were prepared in a cylindrical mold accordingly (2 × 1 × 5 cm). A glass plate was positioned at 25° in an 8 cm container, the scaffold was laid flat on the glass plate, and 6 mL of blood at 37 °C was added to the container. The distance and area of blood uptake were recorded for 5 min. The blood travel distance was calculated to evaluate the scaffold’s ability to absorb blood upward.

### 2.11. Biodegradation Assay in Blood Environment

Blood degradation was assessed following the method by Jiménez-Martin et al. (2022) [30]. Scaffolds were weighed and placed in a 24-well plate. Blood was added to each well to 120% of the scaffold’s maximum blood swelling capacity (600 µL/scaffold). The samples were incubated at 37 °C. At the determined interval, the scaffolds were washed with PBS, freeze-dried, and weighed again. 

### 2.12. Blood Coagulation Assay

Blood coagulation was assessed using the method by Liu et al. (2014) [31]. Scaffolds were placed in a 24-well plate and incubated at 37 °C for 5 min. Whole blood (200 µL) was added to each well. Coagulation was initiated by adding 200 µL of 0.2 M CaCl_2_. The plate was incubated at 37 °C with gentle shaking for varying times (10, 20, 30, 40, and 50 min). 2 mL of deionized water was added, to lyse red blood cells. The absorbance of the resulting solution was measured at 540 nm, with higher absorbance indicating greater clot formation.

### 2.13. Swelling Assay in Blood Environment and Blood Absorption Test

Scaffolds were initially weighed (Wi) and then immersed in 500 µL of blood at room temperature for 2 min, 10 min, 30 min, 60 min, and 24 h. At the time interval, the scaffolds were removed, blotted to remove excess blood, and weighed again (Wf). The swelling ratio was calculated using the formula: Swelling Ratio (%) = ((*Wf* − *Wi*)/*Wi*) × 100, where Wi is the initial dry weight and Wf is the final swollen weight.

### 2.14. Hemolysis Assay

The hemolysis assay was conducted following the protocol by Liu et al. (2014) [31]. Whole blood was collected with citrate tubes. Directly after collecting, whole blood was diluted by adding 8 mL to 10 mL of normal saline. The experimental setup included a negative control, where 10 mL of normal saline was mixed with 8 mL of blood, and a positive control comprising 10 mL of distilled water with 8 mL of blood. Scaffolds were incubated with 10 mL of saline in a tube for 30 min in the 37 °C incubator. After that, 0.2 mL of the diluted blood was added to the tube. After a 60 min incubation period, the samples and controls were centrifuged at 100 g for 5 min, and the supernatant was collected for absorbance measurement at 540 nm. The hemolysis percentage was calculated using the formula: (Absorbance_sample_ − Absorbance_neg_)/(Absorbance_pos_ − Absorbance_neg_) × 100%

### 2.15. Respiratory Epithelial and Fibroblast Cells Culture

Excess human nasal turbinate tissues were ethically acquired with written consent from four Asian patients who underwent turbinectomy. Following procurement, the turbinate tissue underwent thorough washing with Dulbecco’s phosphate-buffered saline (DPBS; Gibco, NY, USA) to eliminate residual blood and mucus. Subsequently, the epithelial layer was meticulously separated from the tissue, minced, and subjected to enzymatic digestion in 0.6% collagenase type I (Worthington, Lakewood, NJ, USA) for 60 min in a shaker incubator set at 37 °C. Upon complete tissue digestion, the resulting sample underwent centrifugation for 5 min at 2370× *g*. The supernatant was then discarded, the pellet washed with DPBS, and subjected to another round of centrifugation for 5 min at 2370× *g*.

The cell pellet was resuspended in a growth medium comprising airway epithelial growth medium (AEGM) (PromoCell, Heidelberg, Germany), defined keratinocyte serum-free medium (DKSFM) (Gibco, Grand Island, NY, USA), and Dulbecco’s modified Eagle’s medium: Nutrient Mixture F-12 supplemented with 5% fetal bovine serum (FBS)(F12:DMEM) (BioWest, Nuaillé, France) in a 1:1:2 ratio. Subsequently, the cells were seeded into a 6-well plate (Thermo Fisher Scientific, Waltham, MA, USA) and cultured at 37 °C in a 5% CO_2_ environment within an incubator.

Medium replenishment occurred every 2 days until the cells reached 80–90% confluency. Following this, the respiratory epithelial cells (REC) and fibroblast co-culture were differentially trypsinized to separate fibroblasts from the culture plate. The culture medium used for respiratory epithelium is AEGM and DKSFM in a 1:1 ratio, while fibroblast was maintained in F12: DMEM + 10% FBS. The medium exchange occurred every 2 days until the cells attained 80–90% confluency, before proceeding to trypsinization at passages 1 (P1) and 2 (P2) for subsequent experiments.

### 2.16. Scaffold Leachate Collection

Leachate was extracted from the sterile scaffold by immersing it in the specified medium at a ratio of 3 cm^2^/mL for 24 h, in accordance with ISO 10993 [32]. The leachate was stored at −80 °C until used.

### 2.17. Cytotoxic Assay

Both cells obtained from respiratory tissue were used in this study (RECs and fibroblasts). RECs or fibroblasts were cultured in their respective 96-well plate until they reached 40% confluency. The cells were then treated with the extracted leachate accordingly. After 24 h of exposure, cell viability was assessed using the 3-(4,5-dimethylthiazol-2-yl)-2,5-diphenyltetrazolium bromide (MTT) assay to evaluate scaffold cytotoxicity.

### 2.18. Proliferation Assay

Cells were seeded in a culture plate accordingly (RECs: 10,000 cells/cm^2^; fibroblasts: 5000 cells/cm^2^). After 24 h, cells were treated with 20% leachate. The treated and untreated cells were observed over 5 days. On day 0, five randomly selected independent fields of the well plate containing the cells were determined and images were captured. These initial points were saved using the NIS-Elements-integrated Nikon microscope software. The same fields were re-imaged daily until day 5. The total number of cells attached to the surface and the proliferation rate were calculated and quantified using the following equation: Proliferation rate (h^−1^) = ln (Total cells attached (final)/Total cells attached (initial))/time).

### 2.19. Wound Healing Assay

Respiratory cells and fibroblast cells were cultured until confluent. Once it was ready, a horizontal scratch was made using 100 µL pipette tips on the plate containing the cells. Leachate was introduced to the cells. Images of the treated and untreated cells were collected for 48 h, and the percentage of closure was calculated using this formula: Wound closure percentage (%) = 100 − (Final area of scratch/initial area of scratch) × 100).

### 2.20. Scaffold Mucoadhesion Assay

In the mucoadhesion study, a simulated nasal fluid (SNF) with a pH of 6.5 was prepared, containing 7.45 mg/mL NaCl, 1.29 mg/mL KCl, and 0.32 mg/mL CaCl_2_ [33]. The experimental setup involved fixing redundant human turbinate mucosa onto a glass slide and submerging it in the SNF. A scaffold was then adhered to the mucosa, and the entire assembly was subjected to stirring to simulate nasal conditions. The time taken for the scaffold to detach was recorded, and the mucoadhesion performance was evaluated based on their adherence duration.

### 2.21. Cumulative Protein Released Assay

Conditioned medium (CM) was collected following the method of Nundisa et al. (2021) [34]. P3 fibroblasts were used for the collection. Once the fibroblasts reached 90–100%, pure F12: DMEM was added to the flask then the CM was collected after 3 days in culture. CM was stored at −80 °C until used. Five samples of CM were collected and pooled together before BCA assay was performed. Scaffolds were fabricated with the additional step of adding 200 µg/mL of CM per scaffold into gelatin and carrageenan mixture. After fabrication, the scaffolds were kept at 4 °C. For the protein release assay, scaffolds without CM and scaffolds with CM were immersed in PBS. PBS was collected daily up until day 7. BCA assay was performed to quantify the amount of protein released in the DPBS. The cumulative protein released graph was plotted by deducting protein concentration released by scaffolds with CM from scaffolds without CM and cumulative daily amount was calculated.

### 2.22. Shelf Life

After scaffold fabrication, all tested scaffolds were kept in an airtight container containing desiccant at room temperature for 3 months. After that, the hydrolytic degradation of scaffolds was evaluated for 18 days.

### 2.23. Statistical Analysis

Experiments were performed in triplicate and repeated on at least three biological samples (*n* = 3). Data are presented as the mean ± SEM. For statistical analysis, one-way and two-way ANOVA were used depending on the variables involved in the comparison. Statistical significance was assessed with Tukey’s multiple comparison. The statistical analysis was performed using Prism 8 (GraphPad Software Inc., San Diego, CA, USA). The results were considered statistically significant at *p* < 0.05.

## 3. Results and Discussion

Nasal packs play a crucial role in various endonasal surgical procedures such as turbinoplasty, septoplasty, and paranasal sinus surgeries. They are inserted into the nasal cavity post-surgery to achieve several objectives, including stopping bleeding (hemostasis after epistaxis), reducing clot formation, lowering the risk of adhesion (synechia) between nasal walls, promoting healing, and providing support to the nasal structure and soft tissue [35,36]. Various materials have been used to manufacture nasal packs for this purpose.

### 3.1. Scaffold Fabrication and Morphological Characterization

In this study, the scaffolds were successfully fabricated and divided into five groups based on their gelatin-to-carrageenan (G:C) ratio: 10:0, 7:3, 5:5, 3:7, and 0:10. Our previous observations show that higher gelatin concentrations resulted in scaffolds with a hard, plastic-like texture and reduced absorption capacity after lypophilization (Supplementary Figure S1). A 1% gelatin concentration was selected as the starting point for its favorable absorption properties. The ability of the sponge to absorb blood is one of the important aspects of the nasal pack.

**Figure 1:**
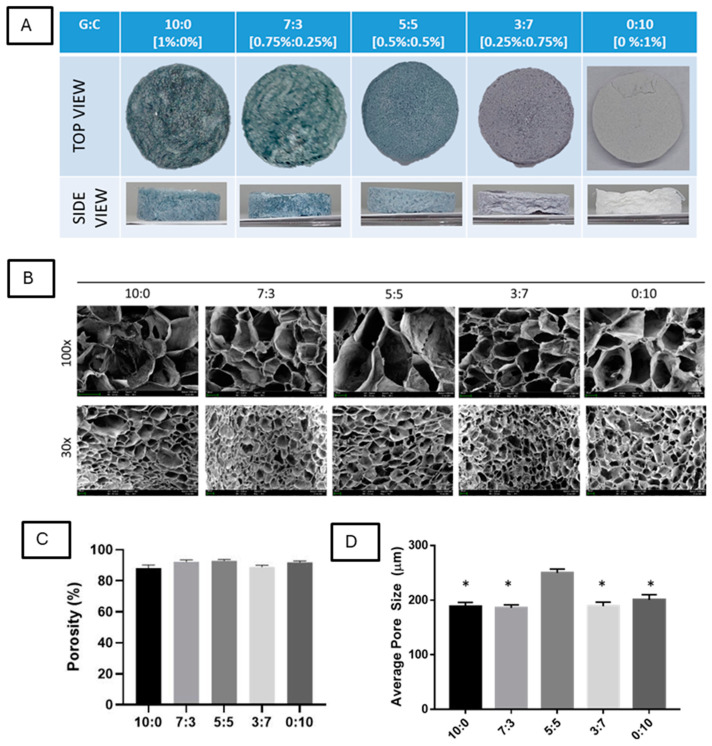
Morphological and Structural Characterization of Gelatin–Carrageenan (G-C) Scaffolds. (**A**) Top and side views of scaffolds with varying G-C ratios (10:0, 7:3, 5:5, 3:7, 0:10), showing genipin-induced blue intensity. (**B**) SEM images (100×, 30×) illustrating scaffold pore structure. (**C**) Porosity (%) across all groups remains consistent at 80–85%. (**D**) Average pore size (µm) (* *p* < 0.05).

Following crosslinking at room temperature overnight, all scaffolds exhibited a blue hue due to the interaction with genipin, with the most intense coloration observed in the 100% gelatin group (10:0). In contrast, the 100% carrageenan group (0:10) appeared colorless. Subsequent lyophilization caused the dark blue color to fade to a lighter shade (Figure 1A). Genipin, a natural crosslinking agent, reacts with the primary amine groups in gelatin to form irreversible crosslinks, leading to the formation of a blue pigment [37,38,39]. This color change is attributed to the formation of covalently crosslinked networks between genipin and the primary amine groups of the lysine residues in gelatin [40]. The shade of the scaffolds is the result of the crosslinking agent, genipin, used during fabrication. While the coloration does not affect the scaffold’s functional properties, it can provide a visual indication of the scaffold’s composition and crosslinking activity.

Scanning electron microscopy (SEM) analysis indicated that the average pore sizes of the scaffolds ranged from 200 to 250 µm following lyophilization. Although slight pore size variations were observed among the groups, all scaffolds maintained sizes within this range (Figure 1B). Porosity measurements obtained through liquid displacement testing showed no significant differences between scaffold groups, with porosity values consistently between 80% and 85% (Figure 1C,D). It was reported that porosity and pore size are critical factors influencing the swelling behavior of scaffolds in tissue engineering applications. The interconnected pores in scaffolds, with pore diameters ranging from 54 to 532 μm and porosities of 66% to 94%, have been shown to influence the swelling behavior of the scaffold [41].

### 3.2. Chemical Composition of Gelatin–Carrageenan Scaffold

**Figure 2:**
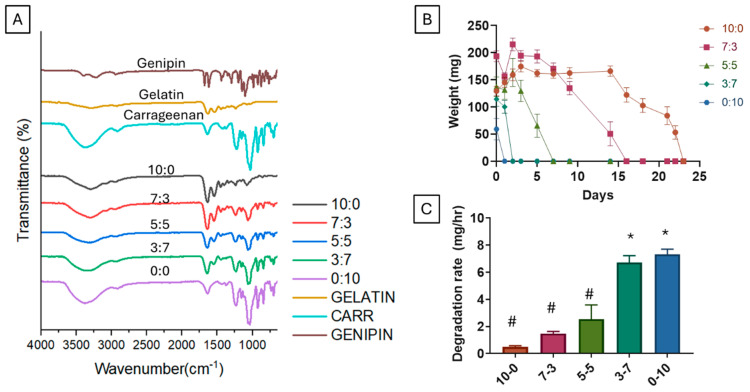
FTIR, Hydrolytic Degradation, and Degradation Rate of G-C Scaffolds. (**A**) FTIR spectra of gelatin, carrageenan, genipin, and scaffold compositions (10:0 to 0:10), highlighting amide and sulfate peaks. (**B**) Hydrolytic degradation over 25 days, with 10:0 showing the slowest and 0:10 the fastest degradation. (**C**) Degradation rates, with 3:7 and 0:10 showing significantly higher rates (* *p* < 0.05 vs. 10:0, # *p* < 0.05 vs. 0:10).

The chemical composition of the scaffolds was analyzed using Fourier Transform Infrared (FTIR) spectroscopy (Figure 2A). The presence of carrageenan in the scaffolds resulted in distinct signals at 840–850 cm^−1^, attributed to D-galactose-4-sulfate, and at 925–935 cm^−1^, indicative of 3,6-anhydro-D-galactose [42]. Additional intense peaks were observed in the 1210–1260 cm^−1^ region, corresponding to sulfate esters (S=O), and in the 1010–1080 cm^−1^ region, associated with glycosidic linkages [42]. In scaffolds containing gelatin, distinctive absorption peaks were observed at 3289 cm^−1^, representing C-H stretches and N-H aliphatic bonds, which shifted to 3370 cm^−1^ upon the introduction of carrageenan. Furthermore, peaks at 1550 cm^−1^ and 1634 cm^−1^ were attributed to gelatin’s N-H bending (amide II) and amine C=O stretching (amide I). The presence of genipin was confirmed by an absorption peak at 1680 cm^−1^, representing C-O stretching vibration, and another peak at 1622 cm^−1^ attributed to C-C vibration of the olefin ring. These results confirm the successful crosslinking and incorporation of gelatin, carrageenan, and genipin in the scaffold.

### 3.3. Degradation Behavior

The degradation behavior of the scaffolds was evaluated in the presence of 0.00006% collagenase type 1 (Figure 2B,C). The scaffolds containing higher concentrations of carrageenan (0:10 and 3:7) exhibited rapid degradation, with complete dissolution occurring within 24 and 48 h, respectively. In contrast, scaffolds with higher gelatin content exhibited a slower degradation rate, with gelatin-only scaffolds taking up to 23 days to fully degrade. Interestingly, the degradation process varied depending on the gelatin ratio; the 0:10 scaffold swelled and formed a thick, watery gel, while the 3:7, 5:5, and 7:3 scaffolds disintegrated into smaller pieces, the gelatin-only scaffolds (10:0) maintained their shape, becoming progressively smaller and thinner over time. This differential degradation behavior underscores the gelatin-to-carrageenan ratio’s role in influencing scaffold stability and dissolution. Numerous factors, including porosity, pore size, composition, swelling, and hydrophilicity, influence scaffold degradation rate. Studies have shown that porosity and pore size regulate the degradation rate and the release of degradation products from scaffolds [43]. However, in our study, we found that, even with similar porosity and pore size, a group with higher concentrations of carrageenan degraded faster than the group with gelatin, which showed that the architecture of the scaffold does not influence the degradation of these scaffolds.

### 3.4. Swelling and Water Absorption Rate of Scaffold

**Figure 3:**
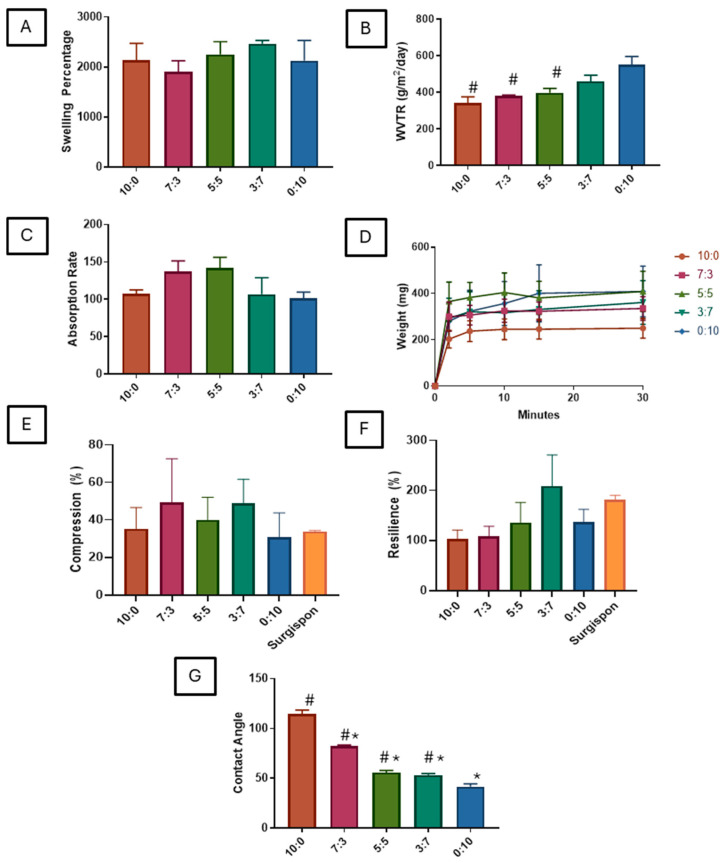
Physicochemical and Mechanical Properties of Gelatin–Carrageenan Scaffolds with Varying Compositions. (**A**) Swelling percentage of scaffolds showing consistent swelling behavior across all groups. (**B**) WVTR, showing reduced permeability in high-gelatin scaffolds (*p* < 0.05 vs. 0:10). (**C**) Water absorption rate of scaffolds indicating similar rates among compositions. (**D**) Water absorption weight after 30 min. (**E**) Compression capacity comparable to Surgispon. (**F**) Resilience percentage reflecting the scaffold’s elasticity, showing comparable resilience to commercial Surgispon. (**G**) Contact angle measurements, indicating greater hydrophilicity with more carrageenan (* *p* < 0.05 vs. 10:0, # *p* < 0.05 vs. 10:0).

All tested scaffolds exhibited significant swelling after 6 h, compared to their initial size, with no notable difference observed between each group (Figure 3A). This finding suggests that scaffold swelling is consistent across gelatin ratios, indicating reliable water uptake. The water absorption rate was evaluated at two-time points: within the first 2 min and at 30 min. Initially, the scaffolds absorbed water at similar rates across all groups, suggesting uniform initial hydration capability (Figure 3C). However, after 30 min, it became evident that scaffolds with higher carrageenan content (e.g., 0:10, 3:7) absorbed more water than those with higher gelatin content (Figure 3D).

### 3.5. Carrageenan Enhances Water Vapor Permeability

The WVTR values of the scaffolds were assessed to determine their capacity to regulate water loss, which is an important parameter for wound dressing applications. The scaffold with the highest gelatin concentration (10:0) demonstrated the lowest WVTR, indicating limited water vapor permeability, which could potentially retain moisture effectively (Figure 3B). Conversely, scaffolds with increasing carrageenan content showed progressively higher WVTR values, with the 0:10 scaffold exhibiting the highest WVTR. This suggests that the incorporation of carrageenan enhances the water vapor transmission, making the scaffold more suitable for applications requiring controlled water loss.

### 3.6. Mechanical Characteristics Comparable to Commercial Nasal Pack

Mechanical stability is crucial for scaffolds intended for tissue engineering. The compression percentage and resilience of the scaffolds were evaluated to understand their mechanical characteristics. The compression testing showed no significant differences between the fabricated scaffolds and the commercial gelatin sponge (Figure 3E), suggesting comparable softness and deformation characteristics. All tested scaffolds were notably compressible with a spongy texture like the tested commercial sponge. Similarly, resilience testing showed no significant differences between the scaffold groups and the commercial control (Figure 3F). The comparable resilience values show that the elasticity of the fabricated scaffolds is suitable for tissue engineering applications, where recovery from deformation is required.

### 3.7. Carrageenan Increases Hydrophilicity of Scaffold

The contact angle measurements provide insights into the surface hydrophilicity of the scaffolds. The results showed that increasing the carrageenan concentration decreased the contact angle, indicating enhanced hydrophilicity (Figure 3G). The scaffold with a 10:0 gelatin-to-carrageenan ratio exhibited the highest contact angle, indicating the least hydrophilic surface among those tested. Conversely, as the proportion of carrageenan increased (e.g., in the 0:10 scaffold), the contact angle decreased significantly, suggesting that carrageenan enhances the hydrophilicity of the scaffold. Hydrophilic surfaces are beneficial in tissue engineering as they can facilitate better cell attachment and proliferation.

Based on a literature review, it was shown that the hydrophilicity of scaffolds has been found to significantly affect the degradation rate [44]. The hydrophilicity of scaffolds facilitates water infiltration, resulting in faster degradation rates [45,46]. It was also shown that higher concentrations of carrageenan have higher WVTR. It is evident that the hydrophilicity of carrageenan is associated with the water vapor transmission rate (WVTR) of carrageenan-based films. For example, Sedayu et al., 2019 reported that the high hydrophilicity of carrageenan is responsible for the increased WVTR of carrageenan-based films [47]. Additionally, Anis et al. (2021) suggested that the relatively lower hydrophilicity of kappa-carrageenan compared to iota-carrageenan is linked to the differences in WVTR findings [48]. These findings collectively suggest that the hydrophilicity of carrageenan is indeed associated with the WVTR, as indicated in the references provided.

Carrageenan is known to be more hydrophilic due to its chemical structure, which contains a high proportion of hydrophilic sulfate groups [49]. These sulfate groups contribute to the overall hydrophilicity of carrageenan, making it highly water-soluble and swellable [49,50]. The hydrophilic nature of carrageenan has been attributed to its ability to form gels in the presence of water, as well as its interactions with other hydrophilic polymers. This increased hydrophilicity of carrageenan is likely to influence its degradation rate, as hydrophilic polymers generally exhibit faster degradation rates due to increased water absorption and susceptibility to hydrolysis. 

### 3.8. Hemocompatibility of Scaffold

The hemocompatibility of the scaffolds was assessed based on blood absorption rate, swelling percentage in blood environments, degradation in blood, blood coagulation tests, hemolysis percentage, and blood uptake assay. These properties are crucial in determining the safety and efficacy of the scaffolds for nasal packing applications, as they need to interact directly with blood and mucosal tissues.

**Figure 4:**
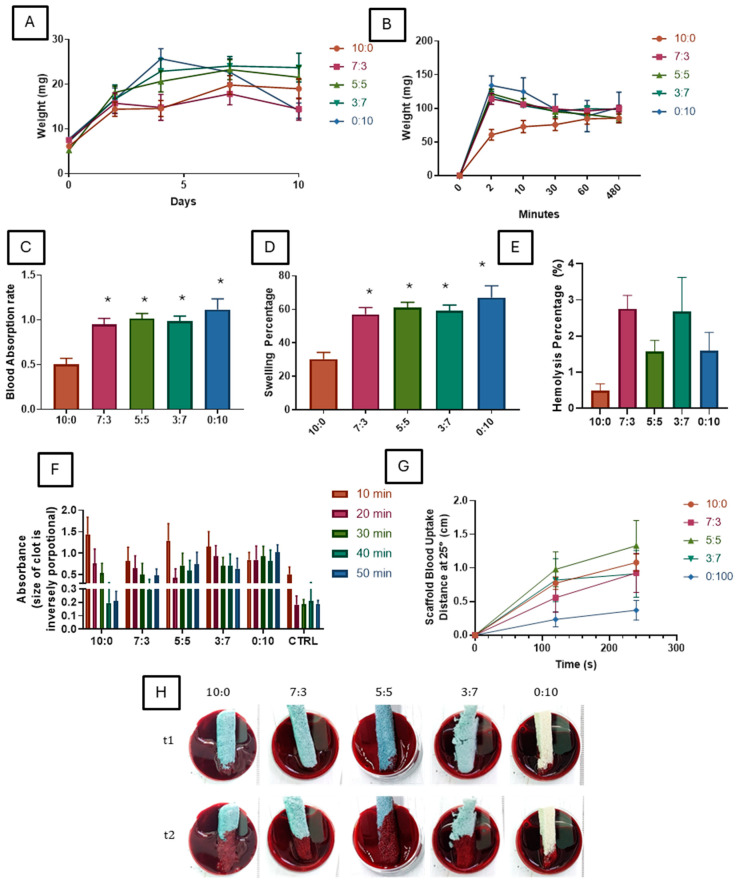
Hemocompatibility and Blood Interaction Properties of Gelatin–Carrageenan Scaffolds. (**A**) Scaffold degradation in blood over 10 days, with faster degradation for carrageenan-rich scaffolds (3:7, 0:10). (**B**) Scaffold weight changes in blood absorption, showing stability after initial uptake. (**C**) The blood absorption rate is significantly higher in carrageenan-rich scaffolds (* *p* < 0.05 vs. 10:0). (**D**) Swelling percentage in blood, higher in carrageenan-containing scaffolds (* *p* < 0.05 vs. 10:0). (**E**) Hemolysis percentage, with a dose-dependent increase in carrageenan-rich scaffolds. Positive control 100%, negative control 0% (**F**) Blood coagulation assay absorbance, indicating slower clotting for carrageenan-rich scaffolds. (**G**,**H**) Blood uptake distance at 25° incline, showing higher uptake in balanced (5:5) scaffolds.

The degradation of the scaffolds in blood demonstrated that scaffolds with higher carrageenan content (0:10) degraded more quickly compared to those with higher gelatin content (Figure 4A). The gelatin-containing scaffold showed a longer degradation time, contrasting with hydrolytic degradation results, where all scaffolds began degrading after day seven. This slower degradation may be due to blood adhering to the scaffold, impeding its breakdown. The faster degradation of carrageenan-rich scaffolds could be advantageous for applications requiring rapid clearance, while the slower degradation of gelatin-rich scaffolds ensures structural stability over a longer duration.

The scaffolds with higher carrageenan concentrations (0:10 and 3:7) exhibited significantly higher blood absorption rates, particularly during the first 10 min (Figure 4B,C). In contrast, the 10:0 scaffold demonstrated limited blood absorption, indicating less interaction with the blood environment. Swelling behavior in the blood was consistent with water-based swelling, where the 0:10 scaffold had the highest swelling percentage, while the 10:0 scaffold showed the lowest (Figure 4D). These findings indicate that carrageenan enhances the scaffold’s ability to absorb and swell in a blood environment, potentially improving the scaffold’s capacity to maintain hemostasis by absorbing excess fluid. In a study by Kasoju and Bora, 2010 they highlighted that sulfation significantly improves the overall hydrophilicity of the scaffold, leading to enhanced blood compatibility [51].

The blood coagulation assay showed that scaffolds containing higher gelatin concentrations tended to promote clot formation over time, as indicated by the decreased absorbance values (Figure 4F). Conversely, carrageenan-only scaffolds exhibited slower clotting activity, which might delay hemostasis. It was shown that gelatin can have both impairing and promoting effects on blood coagulation. Gelatin has been shown to impair blood coagulation by reducing clot strength [52]. However, it has also been demonstrated that gelatin can activate platelets and trigger coagulation, leading to the formation of blood clots [53]. Additionally, gelatin nanofiber sponge has been found to activate the coagulation system in both the extrinsic and intrinsic pathways, promoting erythrocyte aggregation and blood coagulation [54]. Our finding shows that a higher gelatin percentage coagulates blood faster compared to a higher concentration of carrageenan scaffold. It has been established that carrageenans exhibit anticoagulant activity, with λ-carrageenan demonstrating higher potential in this regard compared to κ-carrageenan [55,56,57]. The anticoagulant effect of carrageenans has been attributed to their sulfate groups, which are structurally like heparin, a well-known anticoagulant [58]. Additionally, the molecular weight and sulfate content of carrageenan have been identified as factors directly influencing their anticoagulant activity, with high molecular weight and high sulfate content carrageenan showing greater anticoagulant potential [59,60]. 

Hemolysis analysis demonstrated a dose-dependent response with increasing carrageenan content, indicating greater red blood cell damage with higher carrageenan concentrations. However, hemolysis remained low across all scaffold groups, suggesting an overall hemocompatible behavior (Figure 4E). As per the American Society for Testing and Materials (ASTM), hemolysis levels are categorized as follows: less than 5% is considered null, between 5% and 10% is deemed low, and above 10% is classified as marked hemolysis. Hemolysis refers to the rupture or alteration of the red blood cell membrane, leading to the release of hemoglobin [61]. A previous study that utilized gelatin carrageenan hydrogel at a different ratio as their control group shows that 5% gelatin and 0.5% carrageenan hydrogel ratio gave a reading of below 1% [62]. Our findings indicate that, despite using lower concentrations than those in previous studies, the observed hemolysis levels are below 5% and remain relatively low across the tested group. It is also important to consider the use of the crosslinker genipin in this study. As indicated by another study, the composition of materials, including crosslinkers, can influence and potentially increase hemolysis levels [63].

The blood uptake capacity of various gelatin–carrageenan scaffold compositions was evaluated by measuring the scaffold blood uptake distance over time at a 25° incline (Figure 4G,H). Results indicate that the 5:5 gelatin–carrageenan compositions exhibited the highest blood uptake distance, reaching around 1.5 cm within 240 s. The 10:0 (gelatin-only) and 7:3 compositions showed moderate uptake distances, achieving approximately 1.0 cm, while the 0:10 (carrageenan-only) scaffold exhibited the lowest uptake distance, below 0.5 cm, throughout the assay period. Visual inspection of the scaffolds during blood absorption corroborated these findings, showing extensive blood absorption and spread in the 5:5 scaffolds compared to the minimal spread observed in the 0:10 scaffold. These results demonstrate that balanced gelatin–carrageenan compositions offer superior blood uptake capabilities, suggesting their suitability for applications requiring effective blood absorption.

### 3.9. Fibroblast Cytotoxicity, Proliferation, and Wound Closure

The cytotoxicity, proliferation, and migration behavior of fibroblasts and RECs against the scaffolds were evaluated to assess the biocompatibility and cellular response, which are critical for scaffolds intended for nasal packing applications.

**Figure 5:**
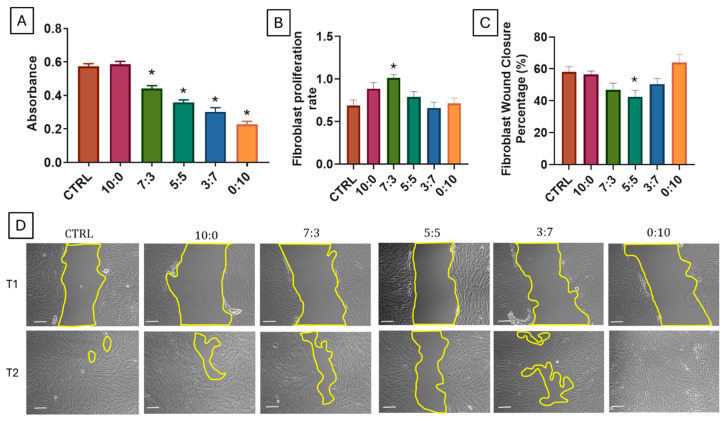
Cytocompatibility and Wound Healing Properties of Gelatin–Carrageenan Scaffolds on Fibroblasts. (**A**) Fibroblast cytotoxicity measured by absorbance, with the highest viability in control and 10:0 (gelatin-only) scaffold, moderate in 7:3, and significantly lower in 5:5, 3:7, and 0:10 (carrageenan-only) scaffolds (* *p* < 0.05 vs. CTRL). (**B**) Fibroblast proliferation rate, peaking in 7:3, lowest in 0:10, showing balanced gelatin–carrageenan compositions support cell growth (* *p* < 0.05 vs. CTRL). (**C**,**D**) Fibroblast wound closure, with highest closure in 0:10, moderate in control and 10:0, and significantly lower in 5:5 (* *p* < 0.05 vs. CTRL). White line scale bar represents 100 µm.

Fibroblast cytotoxicity was assessed by measuring the absorbance of the samples, where higher absorbance values correlate with increased cell viability (Figure 5A). The 10:0 (gelatin-only) scaffold and the control group recorded the highest absorbance, indicating minimal cytotoxic effects and suggesting that gelatin-only scaffolds are well-tolerated by fibroblasts. In contrast, the 0:10 (carrageenan-only) scaffold demonstrated significantly lower absorbance, indicating increased cytotoxicity and reduced cell viability. Intermediate absorbance values were observed for the 7:3, 5:5, and 3:7 scaffolds, which were all significantly lower than the control (* *p* < 0.05), suggesting that incorporating carrageenan reduces scaffold biocompatibility to some extent.

The fibroblast proliferation rate (Figure 5B) was highest in the 7:3 scaffold, indicating that this composition supports fibroblast growth effectively. Research indicates that gelatin improves fibroblast adhesion, migration, and proliferation due to its structural properties and the presence of specific amino acid sequences, such as the RGD (Arg-Gly-Asp) motif, which promotes cell attachment [64,65]. For instance, Nosenko et al. demonstrated that the incorporation of gelatin into fibroin-based scaffolds significantly enhanced the adhesion and proliferation of mouse embryonic fibroblasts, suggesting that gelatin’s presence is crucial for effective cell interaction with the scaffold [66]. Similarly, Xu et al. reported that higher concentrations of gelatin in hybrid biomatrices lead to increased cell-binding motifs, thereby promoting fibroblast adhesion and proliferation [67]. Scaffolds with higher carrageenan content, including 3:7, 5:5, and 0:10, showed proliferation rates similar to the control, suggesting that while these ratios do not inhibit proliferation, they may not actively enhance it.

Fibroblast migration, evaluated through wound healing (Figure 5C,D), showed differing trends compared to proliferation. The 10:0 scaffold and the control group exhibited moderate wound closure percentages, reflecting their general support for migration. However, the 5:5 and 7:3 scaffolds exhibited the lowest wound closure rates, with the 5:5 scaffold significantly lower than the control (* *p* < 0.05). This reduced migration could be attributed to the balanced but lower concentrations of both gelatin and carrageenan, which may not sufficiently support cell movement. In addition, it has been shown that ι-carrageenan hydrogels that have been incorporated with cyclic β-(1-3) (1-6) glucan could increase fibroblast migration [68]. These findings indicate that scaffold composition may have distinct effects on proliferation and migration. While the 7:3 ratio effectively supports fibroblast growth, migration seems to rely on different scaffold properties. 

In this study, we found out that carrageenan causes cytotoxicity at higher concentrations, but in terms of proliferation and migration, cells that are exposed to a higher concentration of carrageenan act similarly to the control. For this, confluency plays a crucial role in cytotoxicity assessments, influencing cell behavior beyond simple cell count or metabolic activity. Gericke et al. (1998) highlighted that confluency provides valuable qualitative insights into cell growth dynamics and responses to cytotoxic agents, particularly in drug screening applications [69]. Heng et al. (2010) demonstrated that higher cell densities significantly altered the cytotoxicity of zinc oxide nanoparticles [70], while Meli et al. (2012) observed that increased confluency in cancer cells was associated with greater drug resistance [71]. Similarly, Lukoseviciute et al. (2023) found that the cytotoxicity of specific inhibitors varied depending on cell confluency, emphasizing the importance of cellular context [72]. Dimanche-Boitrel et al. (1992) further reported that quiescent cells, more prevalent at higher confluency, exhibited reduced sensitivity to cytotoxic agents due to enhanced DNA repair mechanisms [73]. These studies collectively highlight the critical influence of cell density and confluency on cytotoxic responses which explains why carrageenan might cause cytotoxicity in lower concentrations of cells and not in higher concentrations.

### 3.10. REC Cytotoxicity, Proliferation, and Wound Closure

**Figure 6:**
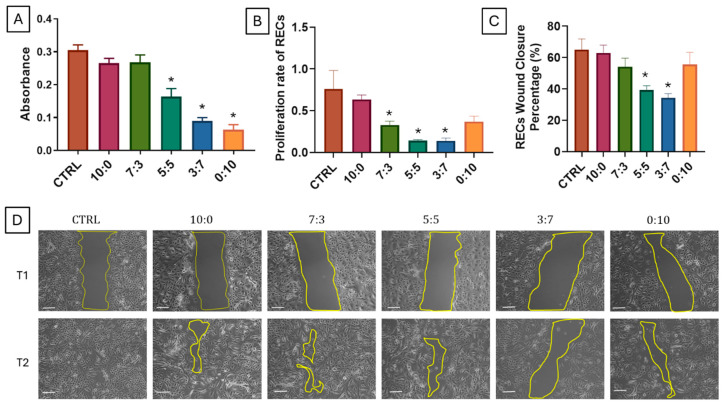
Cytocompatibility and Wound Healing Properties of Gelatin–Carrageenan Scaffolds on Respiratory Epithelial Cells (RECs). (**A**) REC cytotoxicity measured by absorbance, showing highest cell viability in the control and 10:0 (gelatin-only) scaffolds, with significantly reduced viability in 7:3, 5:5, 3:7, and 0:10 (carrageenan-only) scaffolds (* *p* < 0.05 vs. CTRL). (**B**) REC proliferation rate, highest in control and 10:0 scaffolds, with significantly lower rates in 7:3, 5:5, 3:7, and 0:10 scaffolds (* *p* < 0.05 vs. CTRL). (**C**,**D**) REC wound closure percentage, highest in control and 10:0 scaffolds, moderate in 7:3, significantly lower in 5:5 and 3:7, and lowest in 0:10 (* *p* < 0.05 vs. CTRL). White line scale bar represents 100 µm.

Cytotoxicity, proliferation rate, and wound healing percentage for RECs were evaluated across various scaffold compositions (Figure 6). Cytotoxicity was assessed by measuring absorbance levels, with the control, 10:0 (gelatin-only), and 7:3 scaffolds displaying the highest absorbance values, indicating lower cytotoxicity. The 5:5, 3:7, and 0:10 (carrageenan-only) scaffolds showed significantly lower absorbance levels, with 0:10 exhibiting the lowest absorbance, indicating higher cytotoxicity (* *p* < 0.05) in these compositions. For the proliferation rate, the control group exhibited the highest value, followed closely by the 10:0 scaffold. The 7:3 scaffold showed a moderate proliferation rate, while the 5:5, 3:7, and 0:10 scaffolds demonstrated significantly lower rates, with the lowest rates observed in the 5:5 and 3:7 compositions (Figure 6B). The wound closure percentage revealed similar trends, with the control and 10:0 scaffolds achieving the highest closure percentages, followed by the 7:3 scaffold with moderate closure (Figure 6C). The 5:5, 3:7, and 0:10 scaffolds showed lower wound closure percentages, with the 0:10 scaffold exhibiting the least wound closure. 

Both types of cells show similar trends where higher concentrations of carrageenan cause adverse effects to cells. Therefore, the effect of carrageenan appears to be concentration-dependent. However, it has been reported that there is no evidence of cytotoxicity when carrageenan is applied intranasally or by inhalation [74]. In terms of safety, kappa-carrageenan has been evaluated in various studies, confirming its non-toxic nature when used in intranasal formulations. It has been classified as “Generally Recognized as Safe” (GRAS) by the FDA, which supports its use in pharmaceutical applications [75]. Clinical trials have indicated that kappa-carrageenan nasal sprays are well tolerated, with minimal side effects reported among participants [73,76]. Further studies need to be performed to study the effect of carrageenan on airway cells.

### 3.11. Mucoadhesion Study

**Figure 7:**
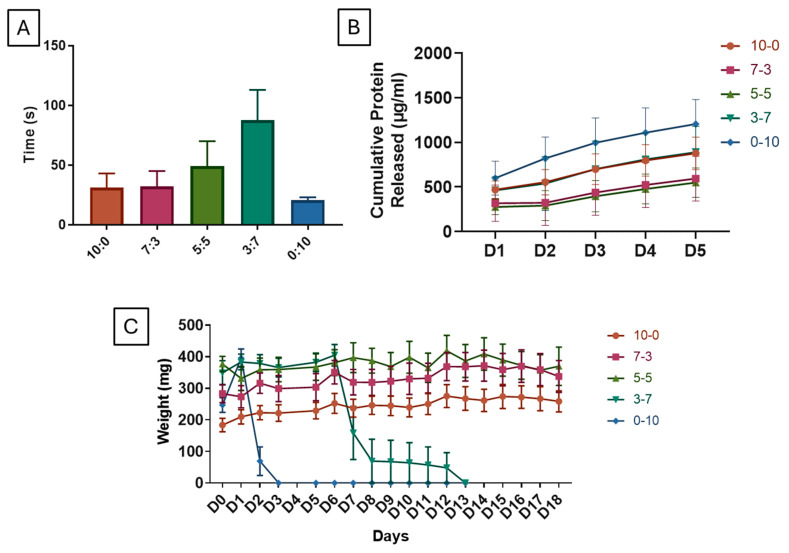
Mucoadhesion, Protein Release, and Degradation Stability of Gelatin–Carrageenan Scaffolds (**A**) Mucoadhesion assay on human nasal mucosa. (**B**) Cumulative protein release over five days. (**C**) Shelf life through 18-day hydrolytic degradation: gelatin-rich scaffolds (10:0, 7:3) were more stable, while carrageenan-rich (3:7, 0:10) degraded faster.

A mucoadhesion study was conducted to evaluate the scaffold’s adhesion to nasal mucosa. In the mucoadhesion study, simulated nasal fluid (SNF, pH 6.5) was prepared and used to evaluate scaffold adherence duration on a turbinated mucosa model (Figure 7A). The carrageenan-only scaffold (0:10) showed the shortest adherence time (20.50 s ± 2.5), while the gelatin-only scaffold (10:0) exhibited moderate adherence (31.00 s ± 12). The balanced gelatin–carrageenan ratios (5:5 and 3:7) demonstrated the longest adherence durations, with the 3:7 ratio achieving the highest mucoadhesive performance (87.50 s ± 25.50). 

The literature provides substantial evidence supporting the mucoadhesive properties of carrageenan in various drug-delivery systems [77,78,79]. Such findings highlight the potential of carrageenan as a versatile mucoadhesive polymer for targeted and sustained drug-delivery applications. However, it needs to be remembered that prolonged usage of nasal packaging is not good as it will promote adhesions. While adhesion remains a concern, it is mitigated by the rapid degradation of carrageenan. These results indicate that a balanced composition is important as it enhances mucoadhesion, which is crucial for maintaining scaffold positioning in nasal applications.

### 3.12. Shelf Life

The shelf life of the scaffolds was assessed to determine the scaffold longevity by evaluating hydrolytic degradation after three months of storage (Figure 7C). The degradation of various gelatin and carrageenan ratios (10:0, 7:3, 5:5, 3:7, 0:10) was analyzed over 18 days. Gelatin-dominant scaffolds (10:0, 7:3) showed stable weight with minimal degradation, suggesting longer shelf life and consistent mechanical stability. The 5:5 ratio offered moderate stability, while carrageenan-dominant scaffolds (3:7, 0:10) degraded quickly. Interestingly, freshly prepared scaffolds degraded faster than those stored on the shelf for three months, indicating a potential stabilization effect during storage.

The tested scaffold has been shown to retain its degradation ability after storing at room temperature for up to 3 months in the dry state. Gelatin is a protein derived from collagen and it is known for its stability and ability to maintain its structure at room temperature, which is why it is commonly used in various pharmaceutical and medical applications, including gelatin sponge scaffolds [4,23,38,39,53]. The stability of gelatin at room temperature is related to its denaturation temperature, which is typically above 30 °C [80]. This means that gelatin maintains its structure and properties below this temperature, making it suitable for storage at room temperature. In addition, in the absence of moisture, the risk of microbial growth is minimized, which helps in maintaining sterility and effectiveness [80]. Meanwhile, when used in sponge form for medical or biotechnological applications, carrageenan is usually stable at room temperature [81]. However, it is important to keep the sponge in a dry, sterile environment to prevent contamination and maintain its integrity. Based on our experience, carrageenan sponges absorb moisture fast from the environment making it wet and soft. This is due to the hydrophilic nature of the carrageenan sponge itself. Therefore, it is important to make sure that the scaffold is in dry environments (e.g., using desiccants in closed containers).

### 3.13. Release Rate

A protein release study examined the scaffolds’ capacity to incorporate protein/secretome, an essential factor in evaluating their drug delivery potential. The cumulative protein release profile from scaffold-conditioned medium (CM) was evaluated over five days (Figure 7B). The carrageenan-only scaffold (0:10) demonstrated the highest release, starting at approximately 750 µg/mL on Day 1 and reaching nearly 1500 µg/mL by Day 5. In contrast, the gelatin-only scaffold (10:0) showed the lowest release, beginning around 400 µg/mL and accumulating to about 650 µg/mL by the end of the study. Scaffolds with balanced gelatin–carrageenan ratios (7:3, 5:5, and 3:7) exhibited intermediate release profiles, with cumulative concentrations between those of the 0:10 and 10:0 scaffolds. The 5:5 and 3:7 compositions showed similar cumulative releases, reaching around 800 µg/mL by Day 5, while the 7:3 ratio displayed a slightly lower release, ending at approximately 700 µg/mL. These results indicate that scaffolds with higher carrageenan content release more protein cumulatively, while higher gelatin content retains protein within the scaffold, resulting in a lower release profile over time. According to Varghese et al. 2014, the presence of higher concentrations of carrageenan leads to a higher release of drugs [82]. Higher carrageenan concentrations in the hydrogel enhance drug release by increasing porosity and hydrophilicity, allowing for easier diffusion of drug molecules. The hydrophilic nature of carrageenan absorbs water, swelling the hydrogel and facilitating faster release. Additionally, higher carrageenan content makes the gelatin–carrageenan network more flexible and promotes matrix erosion, resulting in an extended release over time.

### 3.14. Gelatin–Carrageenan Scaffold as Nasal Pack

In summary, the gelatin–carrageenan scaffolds evaluated in this study demonstrated distinct physical, chemical, and biological properties influenced by their gelatin-to-carrageenan composition. The scaffold compositions with balanced ratios, such as 5:5 and 7:3, showcased optimal characteristics for nasal packing applications, including controlled degradation, favorable hemocompatibility, and enhanced mucoadhesion, all while maintaining structural integrity. The 7:3 and 5:5 gelatin–carrageenan ratios offer a suitable balance of absorption and moisture retention, potentially creating a stable, hydrated environment in the sensitive postoperative nasal cavity. This balance could help reduce the risk of excessive dryness or rapid degradation, which might otherwise interfere with healing. The degradation rates observed for these compositions suggest they may provide effective support over the required timeframe, gradually breaking down as healing progresses. Such controlled degradation could be particularly advantageous in nasal applications, as it may reduce the need for early removal and improve patient comfort.

Additionally, hemocompatibility tests indicate that moderate carrageenan concentrations, as found in the 7:3 and 5:5 ratios, may enhance blood absorption without causing significant hemolysis. This property is beneficial for nasal packing applications, where the material’s ability to absorb blood efficiently is essential for minimizing postoperative bleeding while maintaining a biocompatible environment.

Further supporting the suitability of these compositions, mucoadhesion and protein release assays suggest that the 7:3 and 5:5 ratios may provide adequate adhesion within the nasal cavity, helping to keep the scaffold in place. The controlled protein release profile observed in these assays could be valuable for facilitating therapeutic outcomes, as it allows for a gradual release of bioactive proteins, which might contribute to a more effective healing process.

Lastly, cytocompatibility studies indicate that the 7:3 and 5:5 ratios may support tissue regeneration by demonstrating low cytotoxicity and promoting cell proliferation in fibroblast and respiratory epithelial cell (REC) assays. This biocompatible and supportive environment could help reduce inflammation or irritation, potentially leading to a smoother and faster recovery. Together, the properties of structural integrity, moisture retention, controlled degradation, hemocompatibility, mucoadhesion, and protein release suggest that the 7:3 and 5:5 ratios may be well-suited for nasal packing applications, where stable, gentle support is crucial for effective postsurgical healing.

These results indicate that gelatin–carrageenan scaffolds, particularly in balanced compositions, provide a promising material for nasal packing applications, combining enhanced biocompatibility, tunable degradation, and favorable mechanical and absorption characteristics to improve patient outcomes and treatment efficacy. However, certain limitations should be acknowledged. Firstly, while the study provides detailed insights into the in vitro behavior of fibroblasts and respiratory epithelial cells (RECs) on the scaffolds, the absence of in vivo studies limits the ability to fully predict the scaffolds’ performance under physiological conditions. The complex environment of the nasal cavity, including dynamic mucus production, microbial presence, and mechanical forces, may influence scaffold behavior in ways not captured in this study.

Additionally, although the study thoroughly compared various gelatin-to-carrageenan ratios, the lack of a direct comparison to widely used standard materials, such as cotton gauze or commercial nasal packs, restricts the contextualization of the scaffold’s relative performance in clinical settings. Including such comparisons in future work would provide a clearer benchmark for assessing the scaffold’s utility.

Finally, while this study focused on cellular responses, such as proliferation, cytotoxicity, and migration, other critical factors, including inflammatory responses, extracellular matrix remodeling, and long-term scaffold degradation under physiological conditions, remain unexplored. Addressing these aspects in future research will further validate the scaffold’s clinical potential and enhance its applicability in nasal packing applications.

Building on these findings, future work should focus on in vivo studies to validate the safety, efficacy, and biodegradability of the gelatin–carrageenan scaffolds in clinical nasal packing applications. Evaluating scaffold performance in live tissue environments will provide a clearer understanding of their interaction with nasal mucosa, including their potential to reduce postoperative complications such as adhesions and infection rates. Additionally, exploring the scaffolds’ capacity for delivering therapeutic agents, such as anti-inflammatory or antimicrobial drugs, could enhance their functionality for wound healing and infection control.

Further optimization of the gelatin-to-carrageenan ratio may also refine the balance between degradation rate and mechanical stability, particularly for applications requiring tailored degradation profiles. Lastly, expanding the study to include patient comfort metrics and tissue recovery outcomes will offer valuable insights into the scaffold’s clinical potential, contributing to safer, more effective nasal packing solutions.

## 4. Conclusions

The current study provides significant insights into the physicochemical, hemocompatibility, cytocompatibility, and functional properties of gelatin–carrageenan scaffolds for nasal packing applications. However, further studies are required to validate the long-term clinical efficacy and safety of these scaffolds. In vivo studies will be essential to confirm the hemostatic efficiency, degradation profile, biocompatibility, and overall usability of these materials in clinical settings. Additionally, evaluating the interaction of the scaffolds with nasal mucosa over extended durations, and assessing their performance in different nasal conditions, such as during inflammation or infection, will provide more comprehensive information on their practical application.

The 7:3 and 5:5 gelatin-to-carrageenan ratios demonstrated the most balanced properties, making them the optimal choices for nasal packing based on the evaluated parameters. These scaffolds provide the desired combination of biocompatibility, adequate mucoadhesion, moderate degradation, hemostasis support, and structural stability. With further optimization and validation, these scaffolds hold immense potential as effective nasal packing materials, offering improved patient outcomes in terms of comfort, healing, and reduced complications.

## Data Availability

The original contributions presented in the study are included in the article/Supplementary Material, further inquiries can be directed to the corresponding author.

## Supplementary Materials

The following supporting information can be downloaded at: https://www.mdpi.com/article/10.3390/polym16233387/s1, Figure S1. (A) Top and side views of gelatin–carrageenan (G:C) scaffolds with various ratios and initial stock concentrations, showing differences in texture and morphology. Scaffolds are prepared with five G:C ratios (10:0, 7:3, 5:5, 3:7, and 0:10) across different initial stock concentrations (0.7:0.7, 1:1, 3:2, 6:4, and 10:2% *w*/*v*). Darker shades indicate higher gelatin content, correlating with increased genipin crosslinking. (B) Compression percentage for each scaffold composition compared to Surgispon standard, indicating structural differences among formulations.
