# Supplementary material for: Biodegradable Gelatin–Carrageenan Sponges: High-Potential Functional Nasal Packs for Efficient Secretome Delivery
Source: Polymers (Basel). 2024 Nov 30;16(23):3387. doi: 10.3390/polym16233387 (PMC11644748; doi:10.3390/polym16233387)
Supplement: Supplementary file 1 [file polymers-16-03387-s001.zip › polymers-3318511-supplementary.pdf]

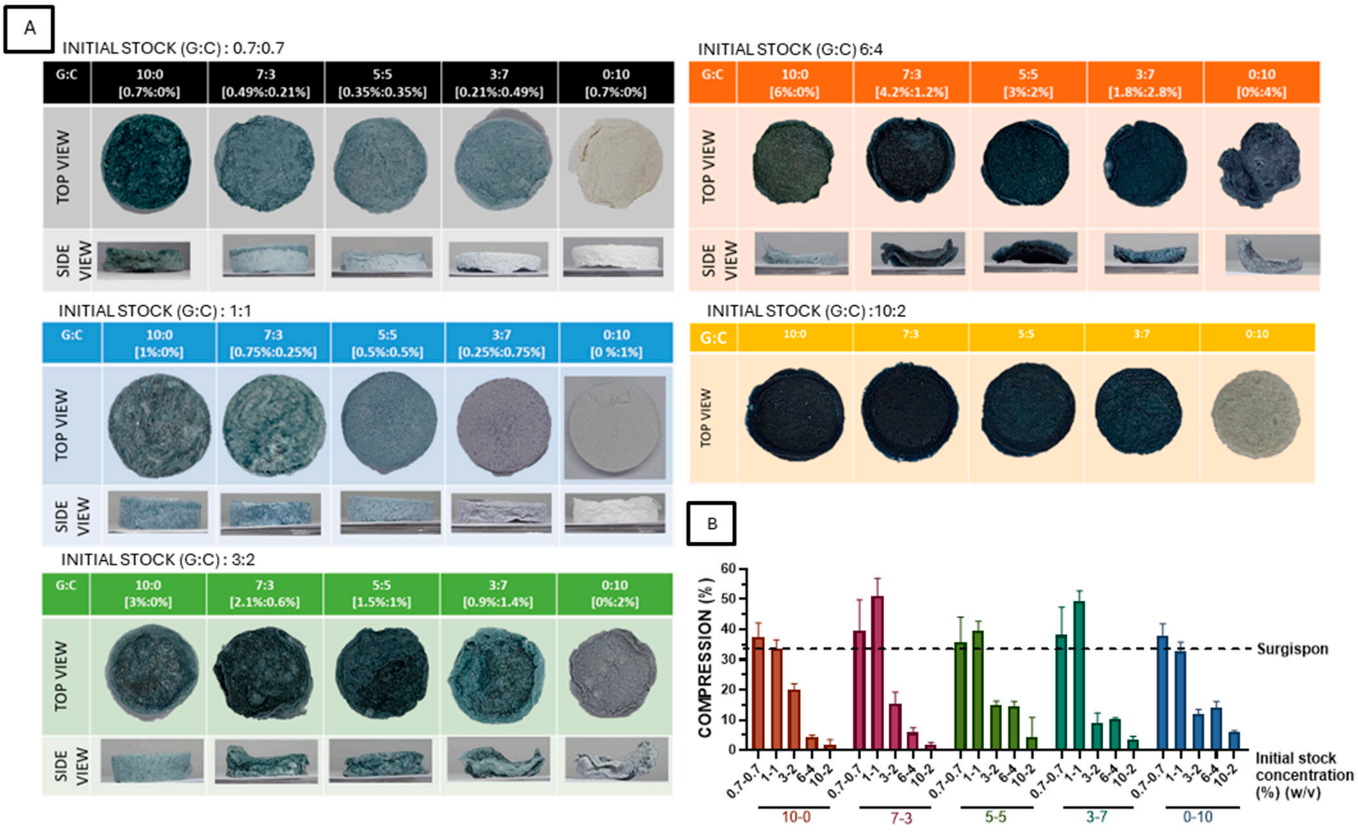

Figure S1. (A) Top and side views of gelatin–carrageenan (G:C) scaffolds with various ratios and initial stock concentrations, showing differences in texture and morphology. Scaffolds are prepared with five G:C ratios (10:0, 7:3, 5:5, 3:7, and 0:10) across different initial stock concentrations (0.7:0.7, 1:1, 3:2, 6:4, and 10:2% *w*/*v*). Darker shades indicate higher gelatin content, correlating with increased genipin crosslinking. (B) Compression percentage for each scaffold composition compared to Surgispon standard, indicating structural differences among formulations.
